# Some Reflections on Boarding School Diet

**Published:** 1912-05-18

**Authors:** 


### THE FEEDING OF SCHOOLBOYS.

### Some Reflections on Boarding School Diet.

(BY AN OLD BOY MEDICO.)

The conference on the feeding of schoolboys has aroused interest in the subject not greater than the matter deserves. The food supplied at boarding schools has improved during the last twenty years, and is incomparably better than it was thirty years ago. About that time the consciences of the schoolmasters were pricked by the loud voiced complaints of the parents. Then was the time when a deal of the income of a house master came out of "meat for breakfast sixpence extra per diem." At three sardines or a slice of cold beef to the portion great was the percentage of profit.

Is it right that the house master's emolument should depend on his genius as a boarding house keeper; that the teacher, guide, philosopher, and friend—in theory—of the pupil should depend for his livelihood on his skill as a marketer; that the profits of a school should be the profits of a private hotel? This is the system. Good or bad, it is not likely yet to be altered. Perhaps the schoolmaster is not the worst teacher, because he is a good man of business. But so long as this is the system short-sighted greed and close cutting of prices will produce schools in which the diet of the scholars is not suitable and sufficient.

What is a sufficient and suitable diet for a school boy? The question cannot be dogmatically answered. Individuals must be considered. It is generally assumed that every schoolboy has the appetite of a hungry hound and the digestion of an ostrich. It is a sort of literary tradition that a schoolboy should delight to gorge himself gluttonously on tarts, sweets, ginger beer, and green apples. No writer on boyhood would forgive himself if he failed to bring out this stale story. Has it not been copied from book to book for generations? Actually many boys are dainty feeders, and many boys who eat heartily suffer much from indigestion. Nor is it the case that boys cannot be trusted not to over eat. The most of them can.

The dainty feeder is repelled by the coarseness and unsavouriness of the "good plain food" put before him at school meals in a fashion that requires robustness of appetite. Without physiological knowledge he satisfies hunger unsuitably at the tuck shop. Had his meal been more tempting he would have saved his stomach and his private pocket. Many boys suffer much from indigestion induced by eating between meals. These boys and the gluttons that cannot be trusted not to gorge to repletion might reasonably put the blame on their silly parents. The first often suffer from physiological sugar hunger, the second from bad education, both the fault of the parents. What is more common than the making of food a matter of reward or of punishment? "Now, Tommy, be a good boy and you shall have a biscuit." "Charlie, you naughty, wicked child! Go to bed at once. You shall have no tea." Who has not heard such as this time and again? But if the child has had food sufficient in quantity and suitable in quality, for the reward to be a delicacy, to eat is to overload his system. The cutting off of his food as a punishment is depriving him of sufficient nourishment for his physiological needs. To make over indulgence a reward is not the way to educate children to temperance.

Suitable feeding will greatly increase the future efficiency of the human race. If mankind followed the example of other mammals breast feeding would be universal and rearing would be a gradual process, the mother's milk being supplemented by increasing portions of adult diet, so from the earliest days the diet would be varied. Variety is one of the first necessities of children's diet. The days of fat mutton, sodden potatoes, and stodgy puddings in the schoolroom are not yet done.

Children not used to be rewarded and punished through their food, accustomed to share in the dainties of the table and not left to gaze with longing eyes and watering mouths on dainty dishes reserved for their elders whilst only things certainly plain and but supposedly wholesome fall to their share, will not grow up greedy. They will have become unconsciously habituated to put food at its proper value, so will eat reasonably from an early age with no other guide than the instinct of hunger. The early Victorian tradition that "plain" puddings are wholesome has probably ruined more careers than drink. How can a young brain wrestle with arithmetic whilst the stomach struggles with suet pudding? As a rule children of all ages dislike fat and like sugary things. There is no physiological reason known for this preference. It exists. Why should it not be indulged? Nature does not give physiological cravings without sufficient cause.

The Puritan belief that what men liked must be bad for their souls, and what men disliked must be good for them was, probably by false analogy, the origin of the custom of insisting on children eating fat and of refusing them sweets. That custom dies hard. Even in this day children seldom get enough sugar with their meals. Sugar as sugar or as sweets or in any form liked by the children should be a large item of school meals. If this improvement were made the trade of the tuck shop would grow less and the bad habit of eating between meals might in time die out. The habit of rushing off to the tuck shop with any pocket money received started in the natural craving of sugar starved boyhood for sweet things. The belief that eating sweets causes bad teeth is a short view. Sweets as part of a meal are wholesome, but the unhealthy condition of the stomach and of the mouth, the result of indigestion, caused by the sucking of sweets and the eating of cakes and pastry between meals, interferes with nutrition and is a cause of decay. Three-fourths of the dyspepsia of boyhood and of adult life comes of the habit of eating between meals. Physiological consideration, good cooking, tasty serving, plenty and variety; these are the things necessary for a satisfactory school dietary.

